# Editorial: Computational aspects of biomolecular recognition and processes

**DOI:** 10.3389/fchem.2024.1405585

**Published:** 2024-04-05

**Authors:** Ambrish Kumar Srivastava, Konda Reddy Karnati, Kshatresh Dutta Dubey

**Affiliations:** ^1^ Department of Physics, Deen Dayal Upadhyaya Gorakhpur University, Gorakhpur, India; ^2^ Department of Natural Sciences, Bowie State University, Bowie, United States; ^3^ Department of Chemistry, Shiv Nadar Institution of Eminence Delhi NCR, Greater Noida, India

**Keywords:** biomolecules, drug-design, computational studies, structures, dynamics

Computational methods have become powerful enough to describe the biomolecular structures and their interactions. A variety of methods have been developed and implemented successfully to study biomolecular systems and processes up to a certain level of accuracy. For instance, biomolecular processes are mediated by protein-protein interactions and protein-ligand interactions. These interactions are studied by molecular dynamics (MD), hybrid quantum mechanics and molecular mechanics (QM/MM) methods. Molecular docking decides how a ligand binds to a protein *in vacuo*. The ligand binding to a protein can inhibit its function and thus, can act as a drug. There are several methods available to find suitable ligands for a particular protein such as virtual screening; structure-property relationship; structure-toxicity relationship; ADMET; etc. Quantum mechanics (QM) offers several first-principal methods to calculate structure; vibrational infrared and Raman spectra; NMR; and UV-vis spectra for biomolecular recognition with a desired level of accuracy. These methods can also be useful in obtaining several parameters, which are closely related to the chemical reactivity and biological activity of molecules. Therefore, the computational aspects of biomolecular studies make an interesting avenue to explore phenomena occurring in living systems, which become of equal importance to several disciplines such as physics, chemistry, biology, medicine, and so forth. Biomolecular recognition or structure determination is closely related to their properties and their interactions govern biomolecular processes. The Research Topic “Computational Aspects of Biomolecular Recognition and Processes” presents a compilation of the latest research and development in this field and comprises six research articles.

Local anesthetics are known for various clinical effects including antioxidant activity and scavenging activity. In order to analyze the effects of aqueous and lipophilic environments, Kavcic et al. performed a combined computational and experimental study using popular anesthetics such as lidocaine, bupivacaine, and ropivacaine. They noticed that all these anesthetics exhibit modest free radical scavenging activity in aqueous environments, with lidocaine demonstrating the highest activity. On the contrary, their antioxidant activity in lipophilic environments appears to be negligible. The authors concluded that scavenging activity is influenced by the lipophilicity of the environment.

The flavivirus NS5, a non-structural protein of the Japanese Encephalitis Virus (JEV) was a serious deadly human pathogen responsible for epidemics in Southeast Asia. It consists of N-terminal methyl transferase (MTase) domain and RNA-dependent RNA polymerase (RdRp). Tiwari et al. explored S-adenosyl derivatives as potential binders with the MTase domain using molecular mechanics and docking studies. Their residue-wise decomposition energy reveals the key hydrophobic residues Gly-81, Phe-133, and Ile-147 in the RdRp-MTase interface. According to them, these residues bind vigorously with S-adenosyl derivatives in the vicinity of the interface between the MTase domain and RdRp.

Wu et al. reported the adsorption of CO_2_ molecules on 3*d*-transition metal (TM) ions doped porphyrins induced carbon nanocone (TM-PICNC) detection in the presence of O_2_ and H_2_O molecules using density functional theory. They found the adsorption energies ranging from 0.03 to −12.12 kcal/mol. The authors noticed that the CO_2_ molecule interacted weakly with Cr, Ni-, Cu-, and Zn-PICNC but strongly with the Sc-, Ti-, and V-PICNC. They concluded that the V-PCNC had high sensitivity to CO_2_ gas, making it a promising candidate for having good sensing ability to CO_2_ gas in the presence of O_2_ and H_2_O.

Lignin, a renewable carbon feedstock, produces p-vanillin, a precursor of protocatechuic acid (PCA) with biofuel potential. In their work, Santos et al., have modified the Cytochrome P450_GcoA_ enzyme by mutating T296àS to enable it to catalyze aryl-O-demethylation of p-vanillin. The study highlights p-vanillin’s flexibility and its alignment with Fe(IV) = O in the active site. The study uses comprehensive MD simulations and hybrid Quantum mechanics/molecular mechanics (QM/MM) calculations to confirm the hydrogen atom abstraction as the rate-limiting step for p-vanillin oxidation.

Joshi and Srivastava, explored the pharmacological potential of flaxseed (Linum usitatissimum) as a medicinal herb, aiming to address the limitations of pharmacological drugs. Through computational and bioinformatics analysis, the study identified target genes of flaxseed associated with various diseases and evaluated their potential therapeutic effects using *in silico* methods. Further, molecular docking and molecular dynamics simulations were performed to assess the binding affinity of flaxseed with three common target proteins (CCDC28b, PDCD6IP, and USP34). Importantly, they identified linseed as safe to use for mutagenic toxicity and other cardiotoxicity measures and unsafe for embryotoxicity, hERG toxicity, and heart failure. Gene Ontology, protein-protein interaction network, and Kyoto Encyclopedia of Genes and Genomes studies indicate that flaxseed could be used as a medicinal herb for the treatment of diabetes mellitus, cardiovascular diseases, inflammatory bowel diseases, and Polycystic Ovary Syndrome.

In another article, Roterman et al., studied a protein folding process that is influenced by various environmental factors, with incorrect pathways potentially leading to misfolded forms. The aqueous environment, cell membrane, and chaperones were considered as components of the external force field. The FOD-M (fuzzy oil drop modified) model was selected that includes the component modifying water environment allowing the assessment of the external force field generated by prefoldin. In this study, prefoldin was treated external force field for actin and tubulin. The analysis of prefoldin structures and its role as an external force field provider sheds light on its impact on the protein folding process, comparable to other environmental factors.

Thus, the Research Topic covers the research articles based on the various aspects of computational research on the exploration of biomolecular systems. We believe that these articles provide a current picture of research trends in this interesting field.

